# Supplementary material for: The sugar content of foods in the UK by category and company: A repeated cross-sectional study, 2015-2018
Source: PLoS Med. 2021 May 18;18(5):e1003647. doi: 10.1371/journal.pmed.1003647 (PMC8171925; doi:10.1371/journal.pmed.1003647)
Supplement: Attachment — Submitted filename: reviewers_response_letter_final.docx [file pmed.1003647.s002.docx]

**Reviewers comments response letter re PMEDICINE-D-20-03288R1**

Thank you to the editors and reviewers for taking the time to review this manuscript. We have taken your feedback on board, particularly in reference to the concerns around missing data and the use of brand-level, rather than product-level sales data. We conducted two sensitivity analyses to address this. We have responded to each comment below, including line numbers referring to the clean copy of the manuscript.

**Notes from the Academic Editor**

Please clearly state the weaknesses and flaws in the nutrient data base collected. In particular in the weights used to give all foods the same weights. This potentially means that obscure foods in one category that are truly niche foods would have the same weight as a popular food item [e.g. a product with no reformulation].

By using weights of sales instead of longitudinally following specific products, this is not true reformulation at all but rather an attempt to examine sugar reductions. This can only be done by looking at each product. Therefore, weighted by product and not by category with the same weights for all. These inherent limitations must be discussed clearly in a revision.

We thank the editor for their comment and agree that this study is looking at sugar reduction and not reformulation. We have rarely used the word reformulation in the paper but have removed it from the keywords section. We acknowledge that sugar reduction is more than just reformulation, it is about products entering and leaving the market, shifting sales between high and low sugar products, as well as changing the sugar content of existing products. We have made this more explicit in the introduction (please see lines 88-90). While this study is limited by its use of a brand-level sales database, looking at total sales does encompass changes in volume sales between high and low sugar products and the entrance and exit of brands from the marketplace, rather than only focussing on the sugar content of existing products over time. The limitations of both the nutrient composition and sales data have been made clearer in the discussion and we have included sensitivity analyses which we hope addresses the editor and reviewers’ comments around individual product heterogeneity within brands, and missing brands (lines 279-309).

**Requests from the editors**

Please revise your title according to PLOS Medicine's style. Your title must be nondeclarative and not a question. It should begin with main concept if possible. "Effect of" should be used only if causality can be inferred, i.e., for an RCT. Please place the study design ("A randomized controlled trial," "A retrospective study," "A modelling study," etc.) in the subtitle (ie, after a colon).

The title has now been updated to: The sugar content of foods in the UK by category and company: A repeated cross-sectional study, 2015-2018

Abstract

- Abstract- please replace “similar period” with the exact timeline

This has been corrected

- Last sentence of the methods and findings must outline 2-3 limitations of your study design/methodology

These have been added

- Please add exact p-values, unless p<0.001 as needed

No p-values necessary

- Please temper all results and conclusions by adding “our results show ..” or similar.

This has been corrected

Conclusions

Please address the study implications without overreaching what can be concluded from the data; the phrase "In this study, we observed ..." may be useful. * Please interpret the study based on the results presented in the abstract, emphasizing what is new without overstating your conclusions. * Please avoid vague statements such as "these results have major implications for policy/clinical care". Mention only specific implications substantiated by the results.

Thank you for your comment. We have removed overarching statements and implications from the conclusion and throughout the paper.

At this stage, we ask that you include a short, non-technical Author Summary of your research to make findings accessible to a wide audience that includes both scientists and non-scientists. The Author Summary should immediately follow the Abstract in your revised manuscript. This text is subject to editorial change and should be distinct from the scientific abstract. Please see our author guidelines for more information: <https://journals.plos.org/plosmedicine/s/revising-your-manuscript#loc-author-summary>

Thank you, an author’s summary has been included (page 3).

Please use square brackets for references and Vancouver style for bibliography

This has been updated

Discussion

- Please begin this section with 1-2  sentences summarising what was done

This has been done, and the first two lines of the discussion (lines 239-241) now read*: “Nutrient composition and food sales data were combined to calculate the sales-weighted mean sugar content of five food categories between 2015 and 2018. The results were compared to PHE’s 5% and 20% sugar reduction targets for 2018 and 2020 respectively.”*

- Several sentences need toning down, for example “Most progress was made in the yoghurt and breakfast cereal categories ..”. I suggest revising to “our results show most sugar reductions were observed in xxx categories” or similar.

We have removed the phrase ‘progress’ throughout and rephrased to ‘greatest reductions’ or similar and removed any overarching statements from the conclusion.

- Line 191- I’m not sure consumer behaviour can be inferred from this data, at least not without major caveats so I suggest removing

The reference to consumer behaviour has been removed and (256-258) now reads: *“This provides insights into how individual companies have reduced the sugar content of their products, potentially as a result of PHE’s call to reduce the sales-weighted mean sugar content of their products by 20%.”*

- Please avoid assertions of primacy and add “to our knowledge” at line 221

This has been done (line 318)

Throughout- while you present the findings as a direct result of PHE voluntary targets, there is no reason to believe that these changes in sugar content were independent of the PHE. I suggest de-emphasizing this aspect of your manuscript and noting that the results “could be” due to PHE directives.

This has been done throughout the discussion and conclusion. We have also noted in the introduction that PHE’s initiative is also part of a wider sugar reduction programme, including the introduction of a soft drinks tax, public health awareness campaigns such as Change4Life, and increased attention on the topic from the mainstream media (lines 92-95).

Please ensure that the study is reported according to the [STROBE] guideline, and include the completed [STROBE or other] checklist as Supporting Information. When completing the checklist, please use section and paragraph numbers, rather than page numbers. Please add the following statement, or similar, to the Methods: "This study is reported as per the Strengthening the Reporting of Observational Studies in Epidemiology (STROBE) guideline (S1 Checklist)."

The STROBE checklist has been added as supporting information and a statement that the guideline has been followed can be found at lines 116-117.

Did your study have a prospective protocol or analysis plan? Please state this (either way) early in the Methods section.

        a) If a prospective analysis plan (from your funding proposal, IRB or other ethics committee submission, study protocol, or other planning document written before analyzing the data) was used in designing the study, please include the relevant prospectively written document with your revised manuscript as a Supporting Information file to be published alongside your study, and cite it in the Methods section. A legend for this file should be included at the end of your manuscript.

        b) If no such document exists, please make sure that the Methods section transparently describes when analyses were planned, and when/why any data-driven changes to analyses took place.

        c) In either case, changes in the analysis-- including those made in response to peer review comments-- should be identified as such in the Methods section of the paper, with rationale.

The following statement has been added to lines 114-116: This study was not conducted as part of any pre-planned analyses. It was undertaken as part of a DPhil (PhD) project with analyses being carried out between March and December 2019. No data-driven changes to the analysis took place.

Reviewer #1

The study by Brandy LK assessed the progress made by UK food companies in sugar reduction in five food categories covered by PHE's voluntary sugar targets (biscuits and cereal bars, breakfast cereals, chocolate confectionery, sugar confectionery and yoghurts) using public available sales data and nutrient composition information of 2515 products under 250 brands nested in 95 companies. They found a 7.5% reduction in total volume of sugars sold per capita and 5.2% in sales-weighted mean sugar content. The sugar reduction was primarily observed in yogurts and breakfast cereals, and more than half the companies did not meet the PHE's 2018 target. This study provided the latest evidence on how the UK food industry responded to PHE's voluntary sugar reduction program and highlighted the issue of accelerating the sugar reduction progress in categories such as confectionery for most UK companies.

The paper is well written. However, there are several points that need further clarification:

1. Can you explain how you generated the percentage (line 152-153) and absolute value (Figure 1) that "reduction in the mean sugar content" or "decrease in volume sales" contributed to the total sales-weighted volume of sugar sold?

The change in the total volume of sugars was calculated based on it being equal to the change in the mean sugar content + the change in the total volume sales of product. This has been added to the methods section (lines 180-183)

2. This study calculated the mean sugar content for each brand by simply averaging out the sugar content of all the product variants (line 98-101). However, the sales might differ greatly among product variants within the same brand (for example, the regular chocolate spread might be more popular than sugar-reduced ones), thus we suggested that the mean sugar content of each brand should be weighted by product-level sales if data available.

Thanks for your comment. We agree that using product-level sales data would be preferable, but it was not available to us, mainly because of the high cost of accessing third-party datasets that collect product-level sales data. We recognise that using brand-level sales data is a significant limitation and have conducted a sensitivity analysis to assess what impact this has had on the results, which is now discussed at length (lines 279-309).

3. Does the Euromonitor covers the sales data of the five food categories in the UK? Can you provide more information about the coverage of Euromonitor sales data?

Euromonitor covers the sales for all five food categories included. More information has been given about the retail channels included in the dataset, and lines 124-127 now read: “*The Euromonitor dataset used in this study includes brands sold through all retail channels, including supermarkets, discount stores, convenience stores, traditional markets and vending machines. It excludes food service. Five categories were identified where the sales database could be directly related to the PHE targets (****Table 1****). All of the sub-categories presented in table 1 were included in this analysis.”*

4. The number and the subcategories listed in table 1 for "sugar confectionery"? are not consistent. Please double check.

Thank you, this has been corrected

5. Please double check the percentage figure of total volume sales for the 62 and 42 brands that can't be matched for their nutrient composite data (line 103 and 106). Should it be 3% in line 103 and 2% in line 106?

Thank you, this has been double checked and is correct. Despite being a smaller number of brands (42), they do represent a slightly larger proportion of total sales.

6. Where does the percentage "five categories which together provide 40-60% of free sugar intake in the UK" (line 175) come from? Can you provide a reference here?

Thank you for your comment, this data was taken from NDNS and a reference has been added.

7. Can you explain more about the potential reason why the %change in sales-weighted mean sugar content between 2015 to 2018 was much smaller in PHE's findings compared to this study? Why the sales weighted sugar content of sugar confectionery category showed a reduction (2.4% [-0.6%, -4.2%]) in this study, whereas there was a small increase (+0.6%) in PHE's report?

We think that this is due to the different data sources used between the two studies. PHE use Kantar data, which is collected by households scanning the barcode of products they purchase. It seems likely that products purchased on impulse and consumed out of the home, such as sugar confectionery, are less likely to be scanned and therefore Kantar data is not capturing the full market. This has been added to the discussion (lines 577-579). It may also be down to the brand level sales data used in this study, where individual product heterogeneity is not truly captured. This limitation has been discussed at length, including running additional sensitivity analyses (lines 279-309).

Reviewer #2: Comments

Generally, I think this is an interesting paper that provides a useful contribution to public health. Reformulation of the food supply is a topic of major interest with regards to population-level strategies to improve diets, prevent obesity, and related NCDs, and in particular it is of great interest whether voluntary pledges vs. mandatory regulations are needed to improve the nutritional quality of the food supply. This paper focused in the UK is useful, especially in comparing and contrasting the changes that have been observed in response to this voluntary initiative compared to UK's mandatory sugary drink tax.

Although I think my comments below are readily addressable, I did find the overall quality of the paper difficult to assess due to some confusion in the methods section. I think that adding some more detail on what exactly was done, as well as the rationale, would help tremendously. I look forward to reading future revisions.

Major queries:

- Clarity around brand-company-product. It was at times difficult to follow what you meant by each term, and they seemed to be used interchangeably in some cases.

Thank you for your comment. The methods section has been re-ordered and the terms brand, product and company have been double checked to ensure clarity. Definitions of product, brand and company are given in 132-139.

- Clarity around sales weighting: providing rationale for why to do the sales-weighting and how exactly it was done (seemingly at the brand level). It could be useful to provide an example.

The rationale for sales-weighting, and a worked example, are now given in lines 147-158.

- Clarity around the sample: did products move in and out? Or you followed specific products over time?

Thank you, we have made it clear that we included all brands including those that move in or out over time (line 137).

- Additional consideration and potentially sensitivity analyses around a) the implications of missing data (e.g. missing categories, other missing data); but also the key limitation that you are weighting at the brand level, which doesn't incorporate the considerable heterogeneity that likely occurs for products within brands. Even if you could do a sensitivity analysis of one specific brand and products within it to show how much variation you might expect to see if one product happened to be sold much more than another product, that would be helpful.

We thank the reviewer for their comments. We have conducted two main sensitivity analyses as suggested (lines 279-309) and have added them to the limitations section of the discussion. We hope that these have addressed concerns around the impact of both the missing data and individual product heterogeneity within brands.

- This article is framed as a pre/post study to address the question, did companies respond to PHE's initiative, but it's impossible to know whether they were already reformulating their products and PHE's initiative just happened to coincide with it. Have you considered adding additional years prior to PHE's announcement?

We appreciate that PHE’s targets sit alongside public health awareness campaigns, including Change4Life, and increased attention from the mass media. We have added a few lines to the discussion to address the fact that the results observed here will not solely be down to this single initiative from PHE and we have made this clear by adding the following (lines 353 onwards) to the discussion: “*It is important to note that PHE’s sugar reduction targets sit alongside a long-running public health awareness campaigns, including Change4Life [9], as well as increased attention from the mass media about the sugar content of everyday products, and therefore results observed are not solely due to merely setting targets.”*

We were not able to add additional years prior to PHE’s announcement as when we acquired the data, only 2 years historical data was available and 2015 was as far back as we could go.

Intro

- It would be useful to link sugar to health, and particular free sugar, as there is a slight but important difference between the concept of free sugar and total sugars, which it seems like PHE is targeting.

We wanted to keep the introduction succinct but appreciate this might mean we lost some context as a result. A few lines linking sugar to energy intake and health have been added to the introduction (lines 76-82).

- You might explain briefly what is PHE for non-UK readers. It would be very relevant to know if this is a government org, ngo, industry-backed group, etc.

Thank you, this is a useful suggestion. PHE is an executive agency of the UK Government’s Department of Health and Social Care and has been added (line 84)

- Consider adding more rationale about why we care if this voluntarily initiative works or not? Particularly in the 2nd to last paragraph, why is it important to understand variability between companies?

Please see response below

- How does this link to global efforts (mandatory or voluntary policy actions) to reduce sugar?

Thank you, we take your point about lack of context and have added the following lines (lines 96-105) to the introduction which we hope address both of your points above:

*“In a time when some countries, states and cities are implementing mandatory nutrition policies, such as taxes on soft drinks (8) and front of pack warning labels (9)(10), there is interest and debate about whether voluntary reformulation measures are effective. The success of this voluntary policy is heavily dependent on action by the entire food industry to reduce the sugar content of their products and to encourage changes in consumer behaviour towards purchasing lower sugar alternatives by launching new product lines or focussing marketing and advertising practices on lower sugar products. It is therefore important to analyse the actions of individual companies in relation to sugar reduction.”*

- Consider more rationale about why it is important to consider sales-weighted data (vs. simply looking at changes in the nutrient composition of products)

We have added this rationale to the methods section and lines 148-150 now read:

*“The data were weighted by sales so that the results indicate what was sold, which is a better indicator of consumption and more relevant to public health than analysing the nutrient content of availability only.”*

- When did PHE actually release their guidelines? It would be useful to understand to provide context for time window that was used in this study.

Thank you, we have added the publication of the targets (March 2017) to line 84

Methods

- Please state what were the other 4 categories you could not analyze? Do you know what % of sales these categories account for? This is an important point, particularly as I noted that beverages were not included at all in this analysis. Was that b/c of Euromonitor or because they weren't included in PHE's initiative? This is important considering all the other sugar reduction efforts going on in beverages.  Also, if this does focus on foods, it would be useful to strengthen in the intro why it is important to consider foods in the diet, not just sugary drinks.

Drinks were excluded from this analysis for two main reasons. The first is that we have previously published a paper using the same data sources and time series that look at the sugar content and volume sales of drinks, in the context of the UK soft drinks industry levy (Bandy et al 2020). Secondly, they are not covered by PHE’s 5% and 20% sugar reduction targets. A comparison of the reductions seen in this study and that presented in our other soft drinks paper is discussed in 369-380

The remaining food categories covered by PHE that we could not align with the sales data represent around 11% of sugar intake for children aged 11-18 whereas the products we have included represent 60% of sugar intake (NDNS, 2018 (7)).

- In your explanation of the brand, it's not totally clear what you're saying. It sounds like Cadbury Dairy Milk and Oreos are considered two separate brands, right?

Yes, the brands listed on line 135 are separate brands and we have now made this explicit.

- Why only consider such a small set of years (2015-2018)? Isn't it useful to consider additional years prior to PHE's announcement to understand if trends accelerated?

We were not able to go further back than 2015 as only two years historical data was available to us (line 144-145).

- With regards to understanding reformulation, did you include products in the analysis that could not be reformulated because they did not contain added sugar originally (e.g. plain oatmeal)?

Yes, all products were included, regardless of whether their mean sugar content changed over time or not. Please note that this study is on sugar reduction, which encompasses changing sales, and not specifically on reformulation.

- Please be clear if Euromonitor was providing brand-level or product-level data. It sounds like it was at the brand level. How did you identify all the product within the brand? Secondly, you note that the data is "sales-weighted," but it is sales weighted at the brand level, right? So if there is variability within the brand by individual product, you won't see that here.

Euromonitor provided brand level data. Composition data was provided at the individual product level, and the two datasets were paired based on name, year and category (methods section, line 147 onwards). We recognise that the brand-level sales data means we will have missed heterogeneity within brands and the product level, and have conducted a sensitivity analysis to assess the impact may have had on the results (line 292 onwards)

- How frequently is the nutrient composition data updated?

This is a repeated cross-sectional study - composition data are annual snapshots taken from supermarket websites on the same date in four years (13^th^ December 2015-2018) (line 144).

- I got confused between brands, products, and companies. For example, in Line 102, you note you couldn't find nutrient data for 62 brands- but in many cases a brand does not equal a product, which is what the nutrient data is on?

Companies manufacture a group of products, which share the same name and are known as a brand, but which includes distinct products (e.g. Mondelez manufacturers different biscuits, including Oreos and Ritz, which are two separate brands). We have edited the methods section, which include definitions of products, brands and companies, and hope that this addresses any confusion.

- It would be helpful to understand the total scope of missing data, at least in the discussion. Sounds like you have about ~17% volume sales missing from missing nutrient data, plus the 4 other categories you could not find in Euromonitor. Would you get a different picture if purchases data were used instead?

It is likely that using product-level sales would give more accurate results than using brand sales, but product-level sales data are not available to us, and these databases also have their limitations. We have discussed the limitations of using brand-level data in the discussion, included conducting sensitivity analyses around missing data (lines 279-291) which we hope addresses the reviewers concerns here.

Analysis: would be useful to understand if brand and company are synonymous. I was a little surprised to see this reported by company when previously you were talking mainly about brands. Also, I'm assuming brands are followed longitudinally over time? Or are these repeated cross sections? What is the sample size?

We have addressed the company-brand-product structure in responses to other comments. We present results in figure 2 by company as companies are the decision making bodies for implementing voluntary sugar reduction initiatives. With regard to the study design, this is a repeated cross-section study rather than a longitudinal panel design. The sample sizes in each year are given in table 2 – over the four years we have data on 7329 products nested in 1407 brands nested in 389 companies.

- I didn't understand the structure of the data. Was it at the product-level, with covariates that included year, category, and company?

This is a purely descriptive analysis, so we did not build any explanatory models. We used the product-level data to estimate mean-sugar content at the brand-level. We then weighted the brand-level data with sales data, and aggregated either to company-level or into food categories and estimated the sales-weighted mean sugar content at each aggregation level.

- Please explain how the sales-weighting by brand level was done in the context of a dataset on products.

A worked example has been given for how the sales-weighting was calculated in 152-158

Results:

- Please include units in table 3

This is stated in the top left-hand corner (tonnes)

- Please include some measure of statistical significance in the text when you describe results (Line 140) <- now line 240-244

Confidence intervals have been added.

- Line 140- the absolute reduction in biscuits was quite similar to that observed in yogurt and cereal, which had higher relative declines mainly because they started out at lower amounts. You might just note this by highlighting the absolute vs. relative change.

Thank you for your comment – we have also highlighted the absolute change here (table 4).

- How did you conduct the analysis that gave rise to the result about product-level changes vs. sales-level changes reported in Line 152-153?

This was calculated assuming the total volume sales in sugar were due to either a change in sales volume or a change in the mean sugar content of products. A paragraph fully explaining this has been added to the methods (180-183)

- Figure 1 is confusing. The bars look as though they represent the total sugar sales in each year, but then the stacked bar in 2018 represents change? I think you would want to find some way to represent that the 1.2 and 0.5 represent sugar that is no longer in the food supply (whereas currently it looks like part of a bar that represents sugar currently observed in the food supply)

We have now applied a patterned texture to the colour fill for the 1.2 and 0.5/grey and orange bars to make the distinction clearer that these are no longer in the food supply.

- On the company analysis, did some companies product products across categories? If so, how did you handle this in analysis?

For clarification, the below line has been added in the results section (229-230) above figure 2: “*Companies that manufacture products in more than one category are presented separately in each relevant category.”*

- Line 164- when parsing out the change due to sugar content and change due to sales, it's not clear why you would want the sugar content to be sales weighted. Isn't the point to show specifically what is going on at the product level, not accounting for changes in behavior (sales)? This is where it is also useful to understand if you only included products that remained in the data over time, since it is unclear how new product entry or product exit affected these numbers (a different concept from reformulation, which is changing the nutrient content of a specific product).

The aim of this study is to assess how the sales-weighted mean sugar content of foods has changed over time (as stated in lines 109-112). This is more than just monitoring reformulation and weighting the data by sales incorporates changes in sales between high and low sugar products, entry and removal of products to the marketplace, as well as the reformulation of existing products. We believe this is more valuable than looking simply at the sugar content of products, without knowing how much they contribute to sugar intake.

- Figure 2: I found it hard to understand whether within each company it has a net increase or decrease in sugar sales. This could benefit from a sentence or two to help the reader interpret what is happening in this figure.

Thank you, we have added a few points to the paragraph above figure 2 to help with its interpretation.

- Worth noting in the discussion that several of the categories that changed most had the smallest levels of starting sugar levels (and particularly in yogurt, some of this is natural sugar).

We thank the reviewer for their comment and have noted this in the discussion (line 366-368).

- Maybe not surprising not to see a reduction in confectionary. Could you discuss how the changes observed might also reflect demand? It seems like if someone is making the choice to have a candy bar, they don't necessarily want it to have reduced sugar levels, whereas this could be a more desired attribute for seemingly healthy products like cereal or yogurt, or even for products that have more variability in sweetness, such as biscuits/cookies.

Thank you for your comments. We have discussed the technical ability and willingness of manufacturers to reformulate different products in lines 369 onwards. There is evidence that the consumer acceptability of reduced-sugar foods is lower than their regular counterparts, therefore manufacturers may be reluctant to reduce the sugar content of products, especially for indulgent products such as confectionery.

Discussion

- Were you able to look at non-sugar sweeteners? (Is this mandatorily reported in the UK)? It would be useful to know if these sugar reductions resulted in a reduction in overall sweetness of the products, or if they were just being replaced with NNS. I would also look more into UK/global trends in NNS. My understanding is that NNS-containing foods are increasing, not just for drinks but in foods as well (in fact, yogurt is a popular category for this).

We were not able to look at sweeteners in this analysis, but are planning some analysis on the use of caloric and NNS in the UK in the future. The nutrient database from Edge by Ascential/Brand View that we have access to includes ingredients information, where NNS are mandatorily declared.

- The paragraph stating line 294- I think it would be more useful to compare to countries that expicitly addressed sugar as part of their policy. In particular, consider the countries that are explicitly including a warning on high-sugar foods. Those are currently: Chile, Peru, Israel; currently being implemented in Mexico and Uruguay. Chile has some results, including a paper on reformulation that was published in Plos Med (Reyes et al)- this may actually be the most relevant comparison for this paper.

Thank you for your comments, we have replaced the French case study with the Chilean prospective study you highlighted here, which we agree is a more suitable comparison (line 400-404).

- Limitations section- it would be useful to discuss more the implications of missing data and the fact that you had to (I think) weight all products within a brand equally. For example, what if one product within a brand accounts for most of the sales in that brand? Like if there was a low-sugar Twix that was developed, for example, but everyone continued purchasing the main Twix, this would show a misleading reduction in sugar for this brand.

Thank you for your comments. We recognise that using brand level sales data over individual product sales means is a limitation. To better understand what impact this limitation has had on the results, we conducted a sensitivity analysis. Lines 300-309 now read:

“*As a sensitivity analysis, we classified each of the 55 brands of breakfast cereals included in this study in 2018 as either ‘product range’ brands, or ‘one product leader’ brands. 45% (n=25) could be classified as a product range brands and 55% (n=30) were one product leader brands . We then compared the mean sugar content of the leading individual product, based on this analysis, with the values used in this study. There was an average variation of 8% between the sugar content of the leading products and the brand values used in this study, representing a difference of 0.5g/100g in sugar content. This sensitivity analysis suggests that the use of brand sales data will not have affected the sugar contents of the majority of brands, but that for a minority it will be a major limitation. Use of individual product level sales would improve this, although datasets that have product level sales data have other limitations (15).”*

- Also talk about how well you can say that these changes were attributable to PHE's target. You didn't use a counterfactual here.

We hope that we have addressed this by discussing other actions being taken to reduce sugar consumption, including public health awareness campaigns in lines 353-356, which now reads: “*It is important to note that PHE’s reformulation targets sit alongside a long-running public health awareness campaigns, including Change4Life (18), as well as increased attention from the mass media about the sugar content of everyday products, and therefore results observed are not solely due to this single policy.”*

- Also consider discussing limitations for what we can understand on public health impact from this study. For example, the effect of sugar reformulation could be greater or lesser for different socio-demographic populations, depending on what brands (and products within the brand), they buy.

This is an important point, thank you. We have added this to the limitations section and lines 311-316 now read: “*The sales data used in this study is for the total UK population and does not breakdown by any sociodemographic factors, therefore any variation the impact of reformulation might have based on income and brand preference could not be determined in this study.”*

Reviewer #3:

This review relates to manuscript PMEDICINE-D-20-03288R1 titled "Assessing progress by UK companies to reduce total volume sales of sugar in selected". The manuscript is well-written and easy to follow.

My main concern relates to the fact that about 20% of data was excluded from the analysis (cf. lines 102-109) without any sensitivity analysis conducted to assess the potential impact of missing data on the results. I would encourage the authors to consider additional analyses under various assumptions about the missing data.

We thank the reviewer for their comment and agree that both the missing data is a limitation here. We have conducted a sensitivity analysis to assess what impact this has had on the results, and lines 279-291 of the discussion now read:

*“However, there are two major limitations of using Euromonitor sales data as here. The first is that Euromonitor groups small and local brands under the umbrella term ‘others’. The ‘others’ brands represented an average of 13.5% of volume sales of the categories included in this study, ranging from 9.3% for breakfast cereals to 21.8% for biscuits and cereal bars. These brands could not be paired with composition data and were therefore excluded from the study. We conducted a sensitivity analysis to try and assess what impact this limitation has had on the results. We assumed that volume sales represented by ‘others’ had the same sales-weighted mean sugar content as the overall category in 2015 and reduced by the same proportion to 2018. The overall sales-weighted mean sugar content of the foods was adjusted taking into consideration these missing brands. The sales weighted mean sugar content of overall foods was 29.1g/100g in 2015, and 27.6g/100g in 2018. This is close to the original results of 28.7g/100g for 2015 and 27.2g/100g in 2018 and suggests that while these missing brands represent a significant proportion of the total volume sales, their exclusion has not had a major impact on this study’s key findings.”*

Minor comments

- On line 33, the abstract mentions 99 different manufacturers but online 42, "50 companies" are mentioned (line 42). Then at the start of the results section (line 128) 95 companies are mentioned. Please clarify/correct as relevant.

While 99 companies are included in the analysis, for ease of presentation, only the top 10 for each of the 50 categories were included in the figures. Line 41 has been corrected and now reads “Of the 50 companies presented in this analysis…”. Line 194 of the results now reads *“…95 companies were included in this analysis (although for clarity, only the top 10 companies are presented in the figures).”*

- It is not always clear which metric is the primary outcome. Given that targets were set for mean sales weighted sugar content, it seems that this should be the primary outcome

Thank you for your comments. Lines 169 now make it clearer that while sales-weighted mean sugar content was the primary outcome, total volume of sugars was also calculated given that it is a marker of consumption and therefore adds important context for public health.

- Please clarify whether all existing sub-categories were included in the 5 categories analysed. For example: 7 sub-categories were included under the "biscuits and cereal bars" category. Are these 7 sub-categories all existing categories under "biscuits and cereal bars"?

All of the included sub-categories were included in the analysis, although these sub-categories were not presented in the results. For clarity, the following line has been added (line 127-128): *“All of the sub-categories presented in table 1 were included in this analysis.”*

- Please provide additional details about the calculation of "sales-weighted mean sugar content" explaining the rationale for weighting the data and the details of the calculation

Thank you for your comment. We have added further explanation and rationale for weighting the data by sales. Lines 148-152 now read:

*“The data were weighted by sales by volume so that the results indicate what was sold, which is a better indicator of consumption and more relevant to public health than analysing the nutrient content of available products only. The composition data were weighted by the brand share to calculate the sales-weighted mean sugar content and total volume of sugar sold by category and company.”*

- Given that the same brands/products were analysed in 2015 and 2018, did the analysis consider the lack of independence ("pairing") between the two periods?

Our analyses are descriptive and we have not built any statistical models that directly compare results in 2015 and 2018. Our estimates of 95% confidence intervals around the percentage change presented in table 4 are based on the confidence intervals calculated for the 2018 data and do not account for uncertainty in the 2015 data.

- Please clarify whether the unit of analysis was the product or the brand.

The unit of analysis was the brand, with mean sugar content of individual products being averaged out at the brand level. We have made this clearer by stating it at line 169.

- Please explain in more details the rationale for weighting the data so that each brand is proportional to total sales and having the sum of the unit of analysis equal to the number of brands.

In studies where food items are the population it can be difficult to decide what is an appropriate unit of analysis. Our analysis is weighted by sales, so it could be argued that each individual sale of a product should be considered the unit of analysis. However, multiple sales of the same product show no variance in the key characteristic that we are measuring (namely the sugar content), because the multiple sales are not of 'unique' products. For this reason, we think it is more suitable to use the brand as the unit of analysis, since it is at the brand-level that all of the variance in our outcome variable is found. Because we want to give greater weighting to brands with higher sales, but do not want to artificially inflate our sample size for uncertainty estimates we rescale sales data so that they all sum to 1 (and hence the unit of analysis equals the number of brands).

- Estimates are presented with confidence intervals as per usual practice; however, it strikes me that the data presented represents the entire "population" of products in the database and is therefore not subject to sampling uncertainty. I am not sure that confidence intervals are particularly meaningful in this case. To give some idea of the variability, I would suggest instead presenting standard deviations and/or quartiles.

We agree that it is not clear whether confidence intervals are appropriate for studies that include the entire population in their sample (as is approximately the case here). Confidence intervals are based on sampling uncertainty and there has been no formal sampling conducted for these analyses. However, when all of the population is included in the sample, this can still be considered as a sample of all possible populations that would arise at different time points (i.e. if we collected our data in November, rather than in December). Ultimately, we prefer to provide confidence intervals as they provide a guide to both the variance within the data and the sample size of the study. So that these two elements can be considered separately, we have also added measures of standard deviation to our results tables.

- I suspect that changes in mean sugar content were only calculated for products present in both 2015 and 2018. Please clarify.

No, the changes in sugar content was calculated for all products present in 2015, and all products present in 2018. This was to capture the changes that would have occurred by products being dropped or added to the market. We have clarified the methods and lines 173-175 now read: “*Change in sales-weighted mean sugar content was calculated between 2015 and 2018, with all products being included regardless if they were not present in 2015 or 2018.”*

- Please consider displaying histograms of % change in sugar content by product for products present in 2015 and 2018.

We thank the reviewer for their comments, and we did consider adding a histogram of % change of sugar content, but as we are not able to track each unique individual product over time, we couldn’t do it. We also considered adding histograms of sugar content of all products for 2015 and 2019 but could we could not see a clear purpose for this, or where it would fit in the results, and so decided to leave it.

- Please consider displaying all 5 categories on Figure 1. In doing so, one could show only the 2018 bar per category since it shows both the 2015 and 2018 levels together with where the change occurred (mean sugar content vs volume sales).

This had been considered prior to submission; however, the changes were so small in some categories that it made the figure illegible and we stuck with the version presented here.

- Figure 2 is a nice way to summarize changes by company. I believe additional text would however help the reader understand the Figure. A complementary figure could be a histogram of Percentage change in the total volume of sugar sold for all companies (without identifying companies)

Thank you, we have added some additional text to help the reader understand figure 2. Lines 225-227 now read: “*Company-specific decreases in total sugar volumes (represented by the black marker lines) were predominately due to reductions in the mean sugar content (represented by the orange bars), although some companies also had reduced sales volumes (represented by the grey bars).”*
