# Supplementary material for: The sugar content of foods in the UK by category and company: A repeated cross-sectional study, 2015-2018
Source: PLoS Med. 2021 May 18;18(5):e1003647. doi: 10.1371/journal.pmed.1003647 (PMC8171925; doi:10.1371/journal.pmed.1003647)
Supplement: Attachment — Submitted filename: Reviewer response letter_final.docx [file pmed.1003647.s003.docx]

**Reviewer response letter:** "The sugar content of foods in the UK by category and company: A repeated cross-sectional study, 2015-2018" (PMEDICINE-D-20-03288R2)

Requests from the editors:

1. Please completely address the comments from the two reviewers.

2. Data availability statement: Thank you for the links to the Euromonitor and Edge by Ascential webpages. For each data source used in your study:

a) If the data are freely or publicly available, note this and state the location of the data: within the paper, in Supporting Information files, or in a public repository (include the DOI or accession number).

b) If the data are owned by a third party but freely available upon request, please note this and state the owner of the data set and contact information for data requests (web or email address). Note that a study author cannot be the contact person for the data.

c) If the data are not freely available, please describe briefly the ethical, legal, or contractual restriction that prevents you from sharing it. Please also include an appropriate contact (web or email address) for inquiries (again, this cannot be a study author).

The data availability statement has now been updated and reads as follows:

This study used data from two commercial sources. The sales data was accessed under licence from Euromonitor International via the Bodleian Library, University of Oxford, using Euromonitor’s database portal Passport GMID. The product information dataset, including nutrition composition data, was purchased for the purpose of the lead author’s DPhil research project from Edge by Ascential. Due to licencing restrictions, the Euromonitor and Edge by Ascential datasets can only be requested from the authors under licence for the purpose of verification and replication of study’s findings via the research group’s Data Access Committee (contact: Trisha Gordon [trisha.gordon@ndph.ox.ac.uk](mailto:trisha.gordon@ndph.ox.ac.uk)). Further use of these datasets must be negotiated with the data owners (Euromonitor contact: Ashton Moses - [passport.support@euromonitor.com](mailto:passport.support@euromonitor.com), Edge by Ascential contact: David Beech - [info@ascentialedge.com](mailto:info@ascentialedge.com)).

3. Abstract: Line 27: Please revise “progress made by UK companies” as it doesn’t seem to reflect the observational nature of the study, and could be rephrased as, “The aim of this study was to assess the sales-weighted mean sugar content and total volume sales of sugar in selected food categories among UK companies between 2015 and 2018” or similar.

Thank you for your comment – this has been rephrased.

4. Abstract: Methods and Findings: Please clarify brand, company, and manufacturer- if any of these terms indicate the same thing, please use consistent terms here and throughout.

We have adjusted the abstract and hope that these terms are clearer. We have corrected the manuscript so the use of the word ‘company’ rather than ‘manufacturer’ is consistent throughout.

5. Abstract: Methods and Findings: Please clearly emphasize that you are noting the key limitations of the study in the final sentence: “The main limitations are that this study does not encompass…”

This has been updated as suggested.

6. Author summary: Under “Why was this study done?” please revise the third bullet point to make it clear that the study is not directly examining and evaluating progress attributable to the targets.

Thanks, this has been corrected and now reads: ‘This study was conducted to see how the sales-weighted mean sugar content of individual companies product profiles have changed between 2015 and 2018.’ (Line 57-58)

7. Author summary: What did the researchers do and find? It might be helpful to mention the sugar content measure was weighted by sales volume.

This section has been updated and now refers to ‘sales-weighted’ mean sugar content

8. Author summary: What do these findings mean? Please remove “...but it’s unlikely the 20% sugar reduction target will be reached by 2020.”

This line (68-69) now reads: ‘Our findings show that there has been a small reduction in the sugar content of foods in the UK and the majority of companies had not met the 5% sugar reduction target by 2018.’

9. Methods: Analysis plan: Thank you for clarifying the study had no pre-planned analysis protocol. However, please indicate all changes to the analysis after the data were examined, such as those arising from peer-review (such as adding sensitivity analyses).

Thank you, this has been updated and this section now reads: *“This study was not conducted as part of any pre-planned analyses. It was undertaken as part of a DPhil (PhD) project with analyses being carried out between March and October 2019. All sensitivity analyses were added during the peer-review process. This study is reported as per the Strengthening the Reporting of Observational Studies in Epidemiology (STROBE) guideline (S1 Checklist).”* (Lines 112-116)

10. Results: Line 194-195: Please clarify what is meant by “top 10” (i.e. in size, in sales volume, etc) in the sentence “...although for clarity, only the top 10 companies for each category are presented in the figures”

This has been updated and now reads ‘although for clarity, only the top 10 companies by volume sales for each category are presented in the figures’ (line 226-227).

11. Discussion: Strengths and Limitations section: The results of sensitivity analyses presented here should be presented in detail in the Results section. More detail would be helpful in classifying the “product range” vs “one product leader” brands, and in addition to the 8% average variation in sugar content for the “leader” product and brand as a whole, the ranges should be presented. Details of how the sensitivity analyses were done should also be included in the Methods.

The sensitivity analyses methods can now be found in the methods section (202-224), including an update on how products were classified, and the results are presented in the results section (lines 274-290) and now include ranges.

12. Discussion: Please re-organize the Discussion as follows: a short, clear summary of the article's findings; what the study adds to existing research and where and why the results may differ from previous research; strengths and limitations of the study; implications and next steps for research, clinical practice, and/or public policy; one-paragraph conclusion.

Thanks, the discussion has been adjusted to follow this order.

13. Discussion: Line 412-413: Please revise to avoid any implication of a causal relationship between your observations and the PHE targets: “In conclusion, our findings suggest there has been a mixed response by companies to the PHE voluntary sugar reduction targets.”

This line has been corrected as suggested and now reads: *“In conclusion, our findings suggest there has been a mixed response by companies to reducing the sugar content of foods in the UK between 2015 and 2018.”* (Line 456)

14. Discussion: Line 415-417: Please revise as the study results do not inform on 2020 data. “Our results show that it is unlikely that the selected categories will meet PHE’s 20% reduction targets by 2020…”

This line has been corrected as suggested and now reads: *“The majority of companies had not met the 5% sugar reduction target by 2018 suggesting that additional policy measures may be needed to further reduce the sugar content of foods.”* (Lines 459-461)

15. Table 3: Please indicate that % change is a comparison between 2015 and 2018

Thanks, this has been corrected

16. Table 4: Please indicate if there is a negative sign missing under percentage change for chocolate confectionery (1.2% vs -1.2%)

Thank you, this has been corrected (-1.2%)

17. Figure 2: Please indicate in the title or x axis label that this reflects 2015 vs 2018.

The title of the figure (now figure 3) reads: “Percentage change in the total volume of sugar sold from the top 10 companies’ product portfolios between 2015 and 2018”

18. References: Please use the "Vancouver" style for reference formatting, and see our website for other reference guidelines: <https://journals.plos.org/plosmedicine/s/submission-guidelines#loc-references>

Reference software (Mendeley) set to Vancouver style – this should now be correct

19. Checklist: Thank you for including the STROBE checklist. Please revise the checklist, using section and paragraph numbers rather than page and line numbers to indicate locations in text.

The STROBE checklist has been updated to sections and paragraphs rather than numbers as requested

Comments from the reviewers:

Reviewer #2: The authors have improved the presentation of results in response to reviewer comments. They are clear about the limitations of using brand-level data in this paper. While this lack of granular product-level data presents limitations, ultimately I do think that because of the challenges and expense of obtaining product-level sales data, with additional clarity in the methods section, this paper could serve as an example to other researchers wishing to analyze category/brand level data using Euromonitor. In addition, this is an interesting and useful paper to understand the extent of sugar reductions in the UK in recent years. A couple of outstanding comments remain:

In my view, the sensitivity analyses are helpful but incomplete. For example- one reviewer pointed out the concern w/missing data (uup to 21.8% for cereal bars). To address this, authors just imputed the same reduction changes as the rest of the category. However, given that these are small and local companies, there is no reason to assume that they would be as able as larger companies to reduce their sugar. A conservative sensitivity analyses would at least assume zero sugar reduction in this group. In addition, it's not clear why you did the sensitivity analyses only for the "umbrella/other" brands and not also the 42 brands for which you couldn't find nutritional data. I don't think simply assuming that the missing brands were the same as included brands is very helpful.

Thank you for suggesting the conservative analysis – this was also suggested by reviewer 3. We have added this scenario to the sensitivity analyses which can now be found in the methods and results sections. The 42 brands for which we could not find data were not sold in the time period given (2015-2018) and were mistakes in the Euromonitor database. When compiling their estimates of sales, Euromonitor analysts use the previous years’ data as a starting point. This means that some brands are carried forward by mistake when they are no longer sold due to human error. Using google and the trade press, the lead author identified these 42 brands as not being sold in the given time period and were excluded from the study. Given that they were database errors, we decided not to include them in the sensitivity analyses. This has been made more explicit in the methods section (lines 162-168): “*42 brands across the 5 categories, representing 3% total volume sales, were human errors in the sales database and were not manufactured in the given time period. As these products could not be matched with corresponding composition data they were removed from the data set”)*. This limitation is discussed in lines 385-386: “*Users of third-party sales databases have no control over the data collection process and there is limited transparency in the methods of data collection, or the reliability of sources [19].”*

Why not also present non-weighted data at the product level? This would be a useful sensitivity check that would allow you to avoid the problem that you can't weight by sales at the product level. Then you can come up with a plausible range of estimates for how much sugar reduction occurred.

In this paper we aim to show sales-weighted changes in sugar levels at company and category-level. Whilst removing the sales weighting would allow us to explore product level variability, it would remove the context in which the products are sold. As a sensitivity analysis, this is not ideal as readers would not be able to ascertain whether changes in results were due to a switch from brand to product level or removal of sales weighting. We have adjusted the sensitivity analyses as suggested by two reviewers (see above) and hope that we now present two plausible sugar reduction scenarios for the ‘others’ category.

Finally, the methods section is clearer, but still could use a bit more work (see detailed suggestions below). I still also did not understand entirely how you decomposed the total changes into sugar reductions vs. behavioral changes (sales), since my understanding is those two components have different units of measure. More detail and perhaps an example could help here as well. This also might benefit from creating a specific "outcomes" section which explains in more detail how outcomes are operationalized.

Thanks for your feedback – we have re-worked the methods, including the addition of a flow diagram, and have responded to your specific points below. With regard to the decomposition of total changes in sugar reductions vs sales volume changes, please see the more detailed explanation below.

Minor:

Abstract

Line 32 Note that these are linked at the brand level

This has been updated

Line 41 Please state earlier what the analysis on companies was and if this was different than from the brand-level analysis.

In response to this and other comments by the editor and reviewers we have reworked the abstract to clarify the distinction between brands, products, companies and manufacturers.

Methods:

In absence of a pre-registered protocol, you should note any analytic changes. These would include sensitivity analyses requested by reviewers, so you can just note that.

Thank you, this paragraph now reads: “*This study was not conducted as part of any pre-planned analyses. It was undertaken as part of a DPhil (PhD) project with analyses being carried out between March and October 2019. All sensitivity analyses were added during the peer-review process.”* (Lines 112-116).

Line 138 what does it mean that the brands were "grouped" by company? Simply that you had an additional variable that was company, or products were at the company level, or that you adjusted your SEs for correlation within companies?

An additional variable in the dataset identified the companies that owned the brands, allowing for analyses to be stratified by company. We hope this has been made clearer in the updated methods section and flow diagram.

The description of products, brands, and companies has improved, and the example in Line 152 is helpful. However, additional clarity is needed to help the reader follow the line of thought. In part, this is because information about how the dataset was created is spread across the methods and data anlaysis section. I recommend starting out with the most disaggregated form of data (product) and then talk step by step how you arrive to the category level. I would keep the data analysis sectoin purely for the actual analysis. Prior to this, you could also create an "outcomes" section that provides detail on the outcomes you used and why (e.g., why sugar vol, the per capita outcome, the sugar-reduction targets).

For example, in Line 147 it is confusing how you mention sales-weighting right away. I think you need to make it explicit here that brands were matched to products at the brand level. Within brand, all unique product types contributed equally (were averaged?). Here is where I would mention that products could fall in or out of the sample, depending on their availability in the market in the respective year (it is confusing to mention this not until Line 174).

And then note that, within a food category, the *brand* level data were weighted by sales.

Finally, it is not clear how the analyses were done incorporating both brands and companies. Did you weight brands within companies, as well as in categories?

You could consider a flow chart as well to help readers visualize this.

Thanks for your feedback. We have re-written the methods and have included a flow chart as suggested, which we now hope makes the data variables and how data were weighted much clearer. An additional variable in the dataset identified the companies that owned the brands, allowing for analyses to be stratified by company. We hope this has been made clearer in the update methods section and flow diagram.

Line 175- how did you use ONS stats to calculate per capita sales? did you just divide sales by # of people?

Thanks for the comment, this has been amended to make it clearer that ONS total population statistics were used to calculate per capita per day values by dividing total volume sales by population size and 365 (lines 176-180).

Line 180- it still is not clear to me how you split the change into mean change in sugar content vs. change in total volume sales. In your formula of total sales, what is the outcome? Expenditures, in dollars or sales? From the figure, it looks like it is in grams (of product? of sugar?). I don't understand how you can add sugar content (presumably in grams of sugar) to change in volume.

We have added more detail about this calculation in the methods and have referenced a previous study that has used a similar technique. The new text (lines 183-192) reads as follows:

The change in total volume of sugars sold was split into the change in the absolute mean sugar content and change in total volume sales using a decomposition formula (Smolina et al., 2012). Briefly, this is derived as follows. Total volume of sugar sold (V) = Mean sugar content (S) * Volume of foods sold (F). Differentiation (to give change in volume of sugar sold over time) gives V’ = F*S’ + S*F’. Let ΔV = V’/V (i.e. the annual percentage change in V). Then ΔV = (F*S’ + S*F’)/S*F = F*S’/F*S + S*F’/F*S = S’/S + F’/F = ΔS + ΔF. Therefore, the annual percentage change in the total volume of sugar sold is equal to the sum of the annual percentage change in the mean sugar content and the volume of the foods sold. This was calculated at the total, category and company level and percentage change was used to attribute absolute changes in the volume of sugar to changes in sugar content and changes in total volume sales.

Sensitivity analyses should be reported in the results section.

Thanks, methods and results sections have been updated accordingly.

Reviewer #3: My previous comments have been adequately addressed. I am just not sure that the sensitivity analysis conducted to account for missing data is sufficient. The authors have assumed the same reduction in sugar content in the 'other' category than overall. I wonder whether a more conservative assumption should be tested as well e.g. by assuming a smaller change in the 'other' category. Otherwise, I would like the authors to provide additional justification supporting the assumption of a similar reduction.

-Laurent Billot

We thank the reviewer for their feedback. Their suggestions for the sensitivity analyses were the same as reviewer 2. We have included this more conservative change as an additional scenario in the sensitivity analyses and this update can be found in the methods and results sections.
