# Supplementary material for: The sugar content of foods in the UK by category and company: A repeated cross-sectional study, 2015-2018
Source: PLoS Med. 2021 May 18;18(5):e1003647. doi: 10.1371/journal.pmed.1003647 (PMC8171925; doi:10.1371/journal.pmed.1003647)
Supplement: Attachment — Submitted filename: response_to_editors.docx [file pmed.1003647.s004.docx]

Response to editors – third revisions

1. Data availability statement: For clarity, please remove “from the authors” from the sentence: “Due to licencing restrictions, the Euromonitor and Edge by Ascential datasets can only be requested from the authors under licence for the purpose of verification and replication of study’s findings via the research group’s Data Access Committee (contact: Trisha Gordon [trisha.gordon@ndph.ox.ac.uk](mailto:trisha.gordon@ndph.ox.ac.uk)).” as it seems that the request would go to the Data Access Committee contact person (importantly, the contact for data access request cannot be the authors of the study).

This has been updated in the appropriate section of the Editorial Manager.

2. Abstract: Methods and Findings: Please briefly describe how sales information in Euromonitor represents all sales in the UK (a fraction of the total sales).

This has been done.

3. Abstract: Methods and Findings: Please mention the total number of companies here: “Of the companies presented in this analysis…”

‘50’ has been added

4. Author summary: Why was this study done? First bullet point: Please change “intakes” to “intake” in the first point.

Done

5. Author summary: What do these findings mean? In the first point, it seems as if this could also be described as “approximately half of the companies” although technically a majority. “...and the majority of companies had not met the 5% sugar reduction target by 2018.”

This has been changed to ‘approximately half’ as suggested.

6. Throughout: For in-text citations, please use square brackets. For multiple references, please do not use spaces within brackets, for example [12,13].

Brackets have been corrected and I hope these are now correct.

7. Methods: Line 138-139: Please briefly describe the fraction of total sales covered in Euromonitor.

Done

8. Methods: Lines 195-196: Please indicate where these results are shown. Please describe results with p values (for example, in Figure 2 or Table 5). “Chi-squared tests were used to test for differences between the mean sugar content of categories in 2018 compared to 2015.”

Thanks for this – the line in the methods was a mistake, as we did not end up presenting absolute mean sugar content (which the chi-squared tests refer to) in the results. We did conduct the Kruskal-Wallis tests for the sales-weighted mean sugar content and the p-values have been added to table 4.

9. Results: Line 276: Please briefly mention again the definition for ‘product range’ brands.

Done

10. Results: Line 284: For the second sensitivity analysis, please briefly mention again the significance of the volume represented as “other” in the analysis. Please briefly mention the two scenarios.

Thanks, this section has been updated.

11. Discussion: Limitations: Line 364: In the paragraph describing Euromonitor data, please mention that Euromonitor does not cover all sales and the implications of this.

This has been done.

12. References: Please check the formatting of each reference. For example, information is missing from #2, 3, and 4. Please check journal abbreviations (for example, PLoS Med in Reference 29). Please use the "Vancouver" style for reference formatting, and see our website for other reference guidelines <https://journals.plos.org/plosmedicine/s/submission-guidelines#loc-references>

This has been done and I hope the references are now correct – Plos Medicine citation style was loaded into referencing software.

13. Table 6: Please provide a legend, with a description that clearly describes what is shown in the table. The table should be able to be interpreted on its own.

Legend for Table 6 has been added under table.

14. Table 7: It may be helpful to include the reference number/citation for this study in the table or legend.

Thanks, reference has been added to table title and column header

15. Figure 3: Please include a descriptive legend for this figure.

A descriptive legend has been added under the figures
